# A Double Mechanism for the Mesenchymal Stem Cells' Positive Effect on Pancreatic Islets

**DOI:** 10.1371/journal.pone.0084309

**Published:** 2014-01-08

**Authors:** Arianna Scuteri, Elisabetta Donzelli, Virginia Rodriguez-Menendez, Maddalena Ravasi, Marianna Monfrini, Barbara Bonandrini, Marina Figliuzzi, Andrea Remuzzi, Giovanni Tredici

**Affiliations:** 1 Dipartimento di Chirurgia e Medicina Interdisciplinare, Università Milano-Bicocca, Monza, Italy; 2 Department of Biomedical Engineering, IRCCS-Istituto di Ricerche Farmacologiche Mario Negri, Bergamo, Italy; 3 Department of Industrial Engineering, University of Bergamo, Dalmine (BG), Italy; Children's Hospital Boston/Harvard Medical School, United States of America

## Abstract

The clinical usability of pancreatic islet transplantation for the treatment of type I diabetes, despite some encouraging results, is currently hampered by the short lifespan of the transplanted tissue. *In vivo* studies have demonstrated that co-transplantation of Mesenchymal Stem Cells (MSCs) with transplanted pancreatic islets is more effective with respect to pancreatic islets alone in ensuring glycemia control in diabetic rats, but the molecular mechanisms of this action are still unclear.

The aim of this study was to elucidate the molecular mechanisms of the positive effect of MSCs on pancreatic islet functionality by setting up direct, indirect and mixed co-cultures.

MSCs were both able to prolong the survival of pancreatic islets, and to directly differentiate into an “insulin-releasing” phenotype. Two distinct mechanisms mediated these effects: i) the survival increase was observed in pancreatic islets indirectly co-cultured with MSCs, probably mediated by the trophic factors released by MSCs; ii) MSCs in direct contact with pancreatic islets started to express Pdx1, a pivotal gene of insulin production, and then differentiated into insulin releasing cells. These results demonstrate that MSCs may be useful for potentiating pancreatic islets' functionality and feasibility.

## Introduction

The transplantation of pancreatic islets currently represents a promising therapeutic option for the management of insulin-dependent diabetes, being an alternative both to the standard therapeutic approach with insulin injections, and to complete pancreas transplantation that has also been proposed [1]. Islet transplantation has the edge over these other therapies since it is a minimally invasive therapeutic approach (versus whole pancreas transplantation), and it gives better metabolic control with respect to insulin administration, thus allowing a reduction in diabetic nervous complications and long-term insulin independence [2], [3].

Despite the encouraging potential, the clinical application of this therapeutic treatment is limited by several key factors such as limited availability, being isolated mainly from cadaveric donors, the poor yield of pancreatic islet explants and, above all, the very limited lifespan of transplanted pancreatic islets which is also a consequence of the immune graft rejection that is only partially dampened by the use of immunosuppressive drugs [3].

In order to improve the feasibility of islet transplantation for the treatment of diabetes, it has been proposed to associate pancreatic islets with Mesenchymal Stem Cells (MSCs), a population of adult stem cells initially identified in bone marrow and then found also in other tissues such as adipose tissue, skin, and amniotic fluid [4]. MSCs are easily harvestable from patients, with high plasticity [5], immunomodulatory properties [6], and with the ability to support cellular survival both through direct contact [7], [8] and by the release of trophic factors [9], [10]. By means of these particular features it can be surmised that MSCs may improve the survival of pancreatic islets and, therefore, the success of the transplantation [11]–[13].

Several *in vivo* studies have demonstrated that, when transplanted with MSCs, a lower number of pancreatic islets is required to allow the glycemic control in diabetic rats, but the mechanisms and the duration of these encouraging results are still under investigation [14]–[16]. Some authors have hypothesized that some trophic factors, such as VEGF [15], CNTF [17], [18], Von Willebrand factor [14]–[17], [19], and Il-6 [20], released by MSCs may be able to prolong islets' survival. Other authors have reported that MSCs can transdifferentiate *in vitro* into pancreatic islet-like cells after prior exposure to chemical compounds [21]–[22] or by genetic manipulations [23]–[25].

Here, we confirmed that MSCs can affect pancreatic islet survival, and we also verified the existence of other important mechanisms, such as the involvement of Pdx1 (Pancreatic and duodenal homeobox 1), also known as insulin promoter factor, a key factor for insulin production and β cell maturation, in the positive effect of MSCs on pancreatic islets.

## Materials and Methods

### Pancreatic Islet Isolation

Male Lewis rats (Harlan Laboratories, Italy) 12 weeks of age were used as donors of pancreatic islets. Animal care and treatment were conducted in conformity with the institutional guidelines, in compliance with national (DL n. 116/1992, Circ. n. 8/1994) and international (EEC Council Directive 86/609, OJL 358, Dec 1987; NIH Guide for the Care and Use of Laboratory Animals, US NRC, 1996) laws and policies. The protocol was approved by the Ethic Committee of Mario Negri Institute for Pharmacological Research (n° BG01/C). All the experiments were repeated at least three times to validate the results.

Islets were isolated from the pancreas of Lewis rats (body weight 250–300 g), using an automatic procedure. Briefly, the pancreas of anesthetized rats were distended with collagenase P solution (Boehringer -Mannheim, Mannheim, Germany), removed and then loaded into a digestion chamber at 37°C. When optimum digestion time was reached, the chamber was flushed with 4°C Hanks' balanced salt solution (HBSS, Gibson Nitrogen Corporation, Paisley, Scotland) and digested tissue was purified by centrifugation on a Histopaque gradient (1.077 g/mL, Sigma, St. Louis, MO). Islets were cultured at 37°C in an atmosphere of humidified air +5% CO_2_ in RPMI 1640 medium (Life Technologies Italia, Monza, Italy), supplemented with 10% fetal bovine serum (EuroClone, Pero MI, Italy).

### MSC isolation and culture

MSCs were obtained from the bone marrow of 10 week-old Wistar rats (Harlan Italy) by flushing the femur and tibia diaphysis with 2 ml/bone of α-MEM to which was added 2 mM L-glutamine and antibiotics. After 48 h the non-adherent cells were removed and MSCs were maintained in α-MEM medium (Lonza Group Ltd Switzerland) plus 20% ES cell screened Fetal Bovine Serum (FBS, Hyclone) [26], and at 37°C in a humidified atmosphere containing 5% CO_2_. Surface antigen characterization was performed with FACSCanto™ flow Cytometer (BD Biosciences, San Josè, CA, USA). For the experiments MSCs were used between passage 4 and 6.

### Direct co-cultures

MSCs were previously stained with DiI red fluorescent dye (30 µg/ml, 1 hour, 37°C, Molecular Probes Inc., OR, USA) and then added to approximately 500 pancreatic islets at a density of 500,000 cells/dish in low adhesion flasks (Corning Inc., NY, USA). Co-cultures were maintained for 4 weeks in completed RPMI 1641 medium. The medium was changed twice a week.

### Indirect co-cultures

MSCs were plated at a density of 500,000 cells/dish onto 35 mm Petri dishes to which were added approximately 500 pancreatic islets placed in a Transwell insert (BD, San Jose, CA, USA). Co-cultures were maintained for 4 weeks in completed RPMI 1641 medium. The medium was changed twice a week.

The ability of MSCs previously stained with DiI to coat pancreatic islets in direct co-cultures was evaluated by examining the cultures weekly under an inverted microscope.

### Assessment of islet viability

Islet survival was evaluated using Calcein AM (BD Bioscience, Franklin Lakes, NJ, USA), a vital fluorescent dye which stains viable cells green. Calcein was added to direct or indirect co-cultures and to pancreatic islets cultured alone (4 µM, 1 hours, 37°C). The cultures were examined under an inverted microscope; viable islets were counted weekly and islet survival percentage was calculated.

### Assessment of pancreatic islet functionality

Pancreatic islet functionality was assessed weekly by analyzing insulin release modifications after variations of glucose concentration in culture medium. Pancreatic islets alone, pancreatic islets in direct co-culture, pancreatic islets in indirect co-culture and MSCs were pre-incubated for 2 hours with fresh complete RPMI medium containing a low glucose concentration (1.67 mM). At the end of pre-incubation, each sample was exposed for 1 hour to medium with a low glucose concentration (1.67 mM), followed by an incubation with medium with a high glucose concentration (20 mM), and finally re-exposed to medium with a low glucose concentration (1.67 mM) for 1 hour. At the end of each treatment the culture supernatants were collected for insulin analysis. The amount of insulin was analyzed using an Enzyme-Linked Immunosorbent Assay (ELISA) kit (Temaricerca S.r.l., Italy) according to the manufacturer's instructions.

### ELISA assay

The supernatants from islet, MSC, and islet-MSC direct and indirect co-cultures were collected at different time points (from 1 up to 4 weeks after co-culture setting), and an ELISA assay was performed to detect the amount of Insulin (DRG Diagnostic GmbH, Germany) and CNTF (Abnova GmbH, Germany) following the protocols provided by the manufacturers. Finally, the reaction was stopped and the optical density of each well was determined at 450 nm within 30 minutes. A calibrator curve with the known concentration of each protein was used and the background levels in normal culture medium were subtracted from each sample for all the factors analyzed.

### Immunofluorescences

The cultures were washed with Phosphate Buffered Saline (PBS) and then fixed in 4% paraformaldehyde for 1 hour at room temperature. Cultures were then washed with PBS, incubated in 20% sucrose for 12 hours and then embedded in Optimal Cutting Temperature (OCT) medium and frozen in liquid nitrogen. Sections of 20 µm were then obtained at a cryostat microtome and immunofluorescence analysis was performed using Insulin (Cell Signaling, 1∶100) or Pdx1 (Cell Signaling, 1∶1000) as primary antibodies according to the manufacturers' protocols. Non-specific binding was blocked with 3% BSA in PBS for 1 hour, and then cells were incubated overnight at 4°C with the primary antibodies. After washing with PBS, the secondary antibodies were incubated for 1 hour at room temperature. Then cells were washed with PBS and coverslips were mounted; the cells were then examined using confocal laser microscopy carried out with a Radiance 2100 confocal microscope equipped with a krypton/argon laser. Noise reduction was achieved by Kalman filtering during acquisition.

### Statistical analysis

Values are expressed as mean± SD of three independent experiments. The statistical analysis was performed using the ANOVA test and Tukey's multiple comparison test with the GraphPad Prism (GraphPad Software, San Diego, CA) statistical package. A *P* value of less than 0.05 was considered statistically significant.

## Results

### Adhesion of MSCs to pancreatic islets

In order to identify MSCs, before setting up the direct co-culture, these cells were stained with the vital red fluorescent dye DiI. When directly added, MSCs were able to coat pancreatic islets thus creating a co-culture system where MSCs could grow in adhesion to pancreatic islets which, on the contrary, remained in suspension in the flask.

**Figure 1:**
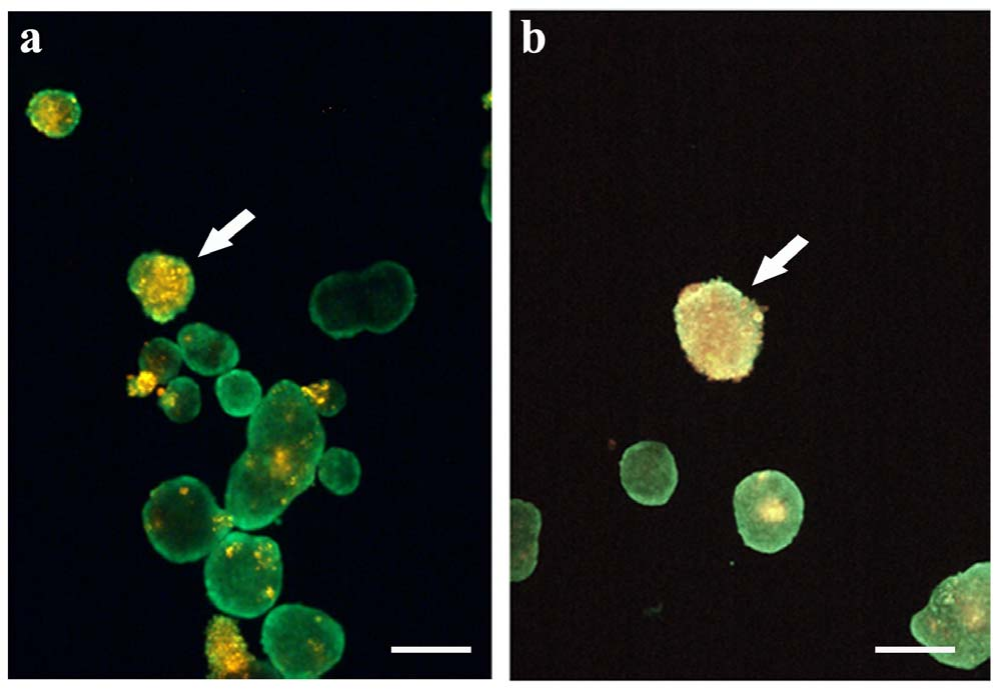
MSC adhesion to pancreatic islets. MSCs previously stained with the red vital fluorescent dye DiI were directly added to green calcein-stained floating pancreatic islets. Attached MSCs are clearly visible as yellow spots (arrows) on green pancreatic islets after 1 week of culture (a) as well as after 4 weeks (b). In green: calcein stained pancreatic islets. In red: DiI stained MSCs. Bar 150 µm.

Most of the MSCs directly added to pancreatic islets (500,000 cells/flask) adhered to the bottom of the flask, where they took the particular fibroblastic-like form already described [6], while a few of these cells adhered to floating pancreatic islets. MSCs attached to the flask were discarded by moving the cellular suspension into a new flask which, therefore, contained only MSC-coated pancreatic islets, as shown in Fig. 1 where MSCs appear as red spots on floating pancreatic islets. The MSC coating of pancreatic islets lasted up to at least 4 weeks of culture, the time point at which we stopped our experiments (Fig. 1).

### Survival of pancreatic islets coated with MSCs

**Figure 2:**
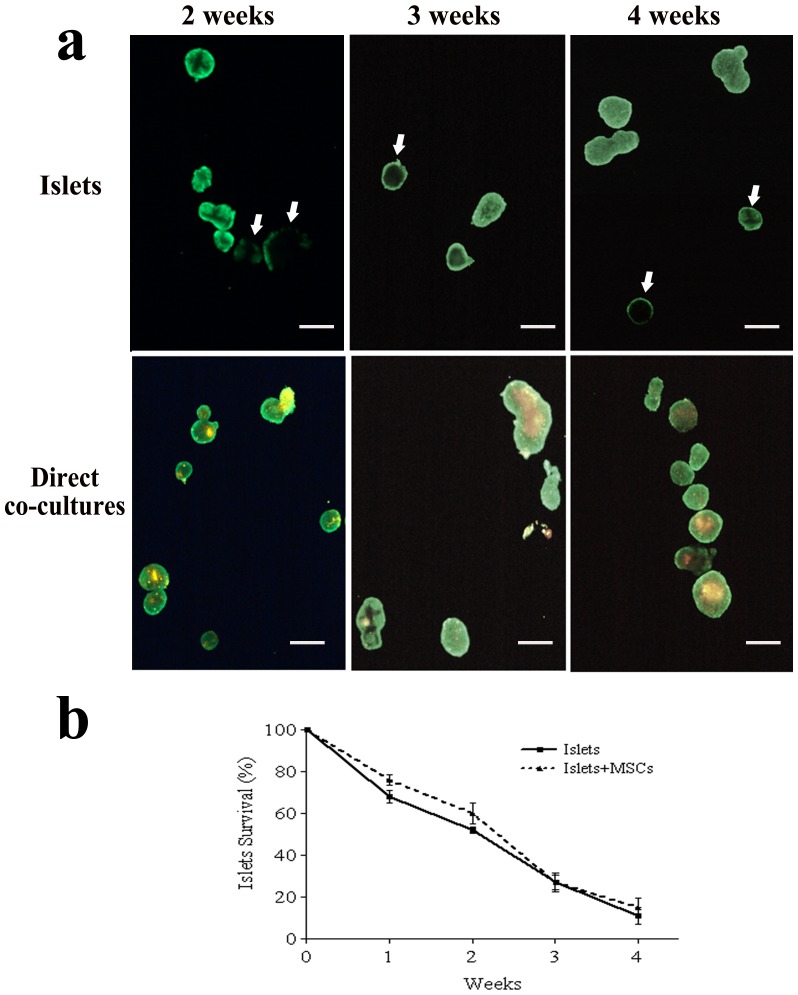
Viability assessment of MSC-coated pancreatic islets. The viability of pancreatic islets coated with MSCs was assessed by using the vital fluorescent dye calcein, which evidenced in green only viable cells. Upper panel: pancreatic islets cultured alone stained with calcein after 2, 3 and 4 weeks of culture. Lower panel: pancreatic islets directly co-cultured with DiI red-stained MSCs which appear as yellow spots after 2, 3 and 4 weeks of culture. Arrows indicate pancreatic islets in which calcein did not spread uniformly. Bar 150 µm. b) Percentage of pancreatic islets' survival, expressed as mean ± SD. Calcein-positive pancreatic islets were counted and the survival percentage was calculated up to 4 weeks of culture.

Calcein, a vital diffusible fluorescent dye that stains viable cells, was used to verify the effect of MSC coating on pancreatic islets' viability. Pancreatic islets cultured alone and co-cultures of pancreatic islets and MSCs were stained with calcein, which evidenced in green the living cells, and were then examined under an inverted microscope at different time points (2, 3 and 4 weeks). As shown in Fig. 2a, calcein had a uniform spread in islets co-cultured with MSCs up to 4 weeks while, in many islets cultured alone, the dye distribution was limited to the cells of the outer rim. Also red MSCs which coated the islets were still viable and were stained with calcein, thus appearing as yellow spots.

The count of viable islets, however, did not evidence any differences between islets cultured alone and those co-cultured with MSCs, since both cultures had a comparable progressive decrease in number, up to 80% after 4 weeks of culture (Fig. 2b).

### Insulin release after glucose stimulation

**Figure 3:**
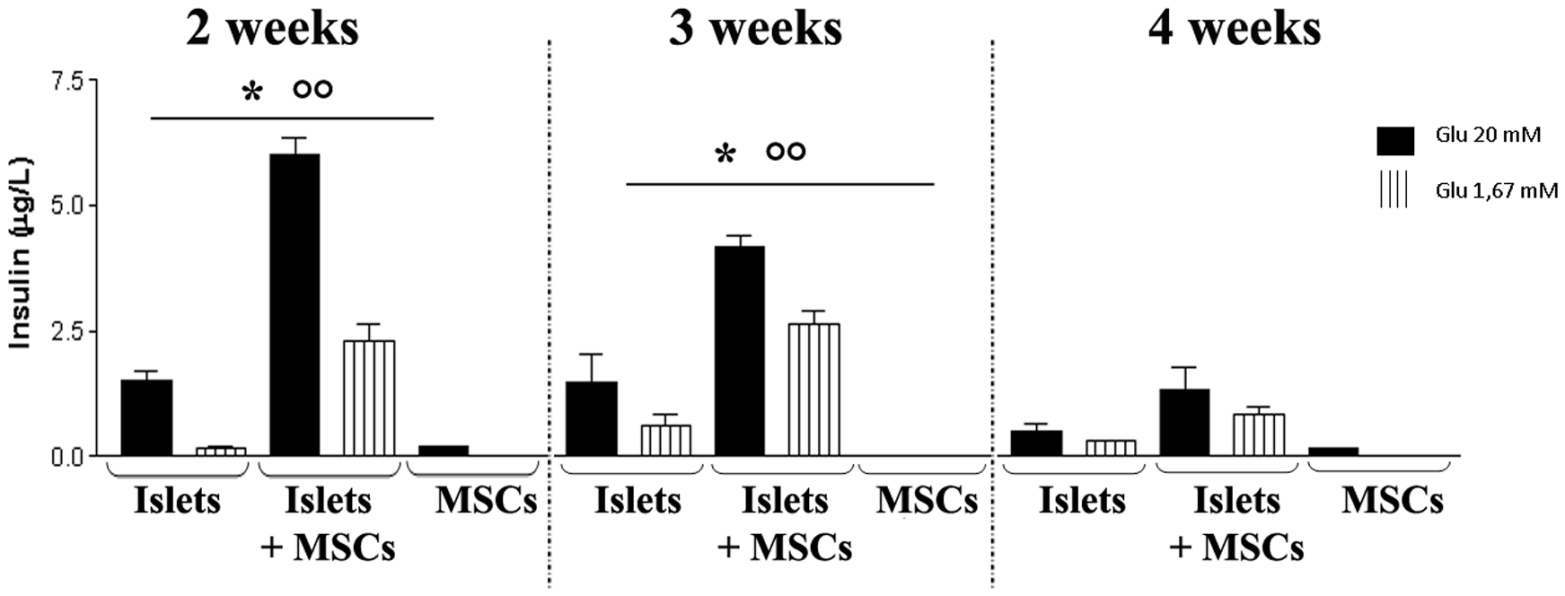
Insulin release after glucose stimulation. Each week (up to 4 weeks of culture) pancreatic islets cultured alone or directly co-cultured with MSCs, and MSCs cultured alone were exposed to different glucose concentrations in the culture medium (20 mM and 1,67 mM Glucose), and the insulin release after each change was measured by an ELISA assay specific for this hormone. Results are expressed as mean ± SD of three independent experiments. * *P*<0.05 Islets vs Islets+MSCs, ^○○^ *P*<0.01 Islets+MSCs vs MSCs.

The effect of MSC coating on pancreatic islets' ability to modulate the insulin release in reply to glucose variations in the culture medium was examined by ELISA assay. As shown in Fig. 3, pancreatic islets were able to adjust the insulin release to the amount of glucose in the culture medium, with a similar variation both with and without MSCs until 3 weeks of culture. After 2 and 3 weeks, the amount of insulin released was, however, statistically significantly higher (at least 3 times) in the culture medium of direct co-cultures of islets and MSCs with respect to the medium of islets cultured alone (Fig. 3). After 4 weeks of culture the low number of MSCs probably affected the release of insulin, and the difference between islets cultured alone and direct co-cultures, although present, was not found to be statistically significant.

The insulin release by MSCs cultured alone in the same culture medium as pancreatic islets was negligible (Fig. 3).

### Insulin expression in direct co-cultures

**Figure 4:**
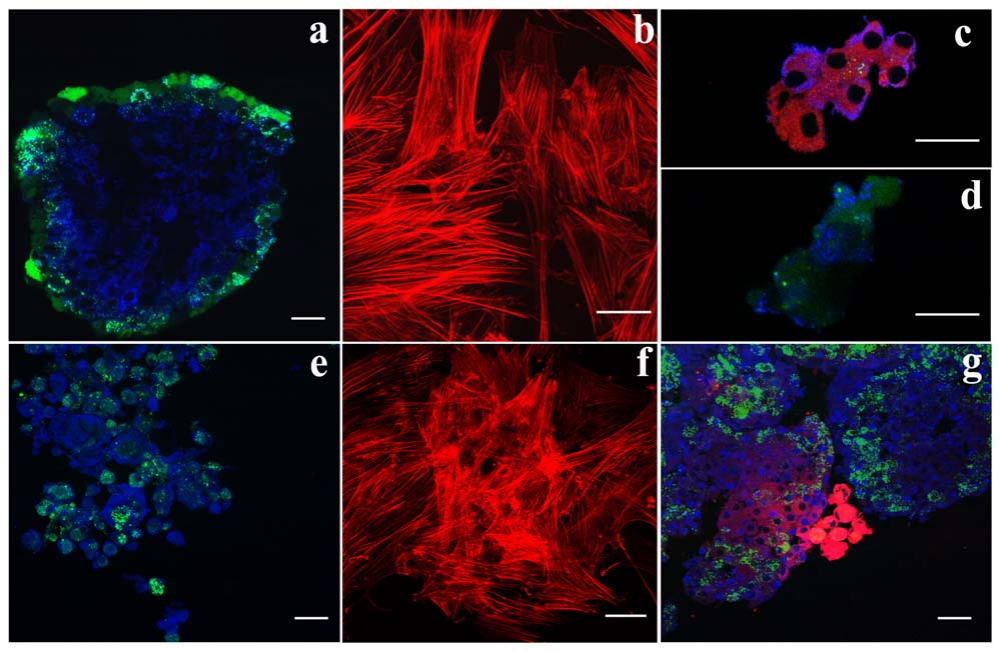
Insulin and Pdx1 detection. Insulin positive cells in pancreatic islets (a), in MSCs (b), and in direct co-cultures of pancreatic islets and MSCs (c and d). In green, calcein positive pancreatic islets. In red, MSCs stained with DiI. In blue, insulin positive cells. Bar 30 µm. Pdx1 positive cells in pancreatic islets (e), in MSCs (f), and in direct co-cultures of pancreatic islets and MSCs (g). In green, calcein positive pancreatic islets. In red, MSCs stained with DiI/Phalloidin. In blue, Pdx1 positive cells. Bar 30 µm.

In order to shed light on the increased release of insulin in the medium of pancreatic islets cultured with MSCs, the distribution of insulin within the islets was examined by immunofluorescence, comparing pancreatic islets alone and in co-culture with MSCs. To allow their identification, MSCs were previously stained with the red fluorescent dye DiI and then added to pancreatic islets which were, on the contrary, stained green with calcein. As shown in Fig. 4, insulin was uniformly spread within the pancreatic islets cultured alone (a), while it was absent in MSCs cultured alone (b), confirming the results of the ELISA assay. In direct co-culture samples there was evidence, as expected, of green-stained pancreatic islet cells positive for insulin (Fig. 4c) but, surprisingly, there was also the presence of red-stained MSCs which had lost their classic fibroblastic-like morphology, taking on a round shape and above all producing insulin (Fig. 4d). The round-shaped MSCs were organized in clusters, very similar to pancreatic islet cells, thus suggesting a progressive differentiation of these cells towards an insulin-releasing phenotype, according to the increased amount of this hormone observed at the ELISA assay (see Fig. 3).

With immunofluorescence Pdx1, a pivotal protein for insulin production, was expressed both by islets and the covering MSCs in direct co-cultures (Fig. 4e and g), but it was otherwise absent in MSCs cultured alone (Fig. 4f).

### Indirect co-cultures

**Figure 5:**
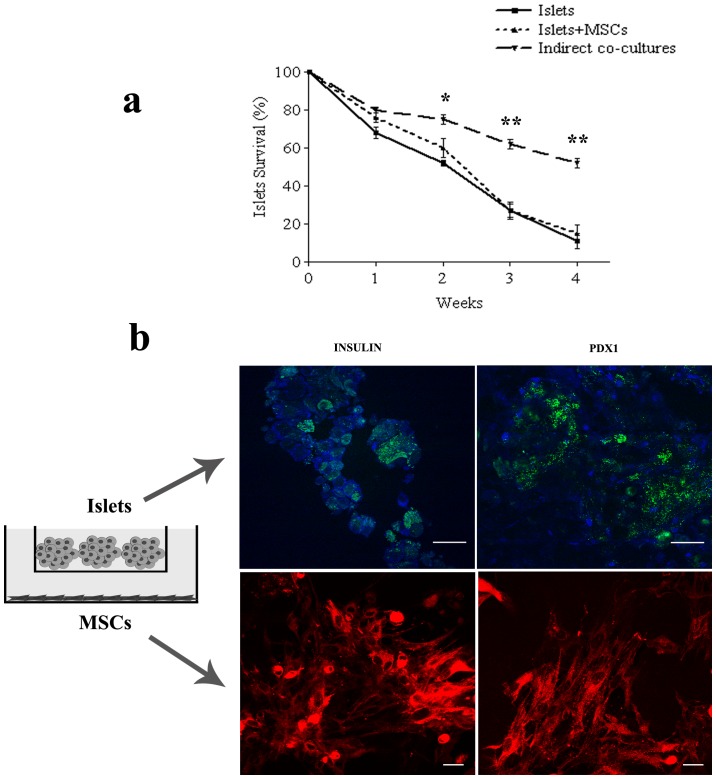
Indirect co-cultures. Survival percentage evaluation of pancreatic islets indirectly co-cultured with MSCs. Calcein-positive pancreatic islets were counted and survival percentage was calculated up to 4 weeks of culture. Results are expressed as mean ± SD of three independent experiments *P<0.01 Indirect co-cultures vs Islets, ** *P*<0.001 Indirect co-cultures vs Islets and Indirect co-cultures vs Islets+MSCs. Insulin and Pdx1 positive cells in indirect co-cultures made up by pancreatic islets (within the Transwell, upper panel), and MSCs adherent to the cover glass (lower panel). In green, calcein positive pancreatic islets. In red, MSCs stained with DiI. In blue, insulin or Pdx1 positive cells. Bar 30 µm.

In order to ascertain whether the differentiation of MSCs into an insulin-releasing phenotype was due to their direct contact with pancreatic islets rather than to some trophic factors released by the islets into the culture medium, indirect co-cultures of pancreatic islets and MSCs were set up. MSCs were attached to the bottom of the flask, whilst floating pancreatic islets were physically separated by the presence of a Transwell which allowed the sharing of the same medium by the two cellular populations. The pancreatic islets, also in indirect co-cultures with MSCs, were still able to modulate the release of insulin according to glucose variations in the culture medium (data not shown). Differently from the results obtained with direct co-cultures, after 3 and 4 weeks the count of viable pancreatic islets evidenced a statistically significant increase in survival percentage (from 20% to 50%) in indirect co-cultures with respect to both the direct cultures and islets cultured alone (Fig. 5a). By immunofluorescence, the MSCs of indirect co-cultures were positive neither for insulin nor for Pdx1 (Fig. 5b).

The important role of some trophic factors such as VEGF and Von Willebrand factor on MSC-mediated islet survival has been already described in the literature [14]–[17]. On the contrary, there are poor results for CNTF, a trophic factor able to increase the islets' viability when exogenously administered [18]. In order to further investigate its role in MSC-dependent promotion of islet survival, CNTF expression and release was studied in all the co-culture paradigms. CNTF was expressed in all the samples, but released in an undetectable amount (data not shown).

### Mixed co-cultures

**Figure 6:**
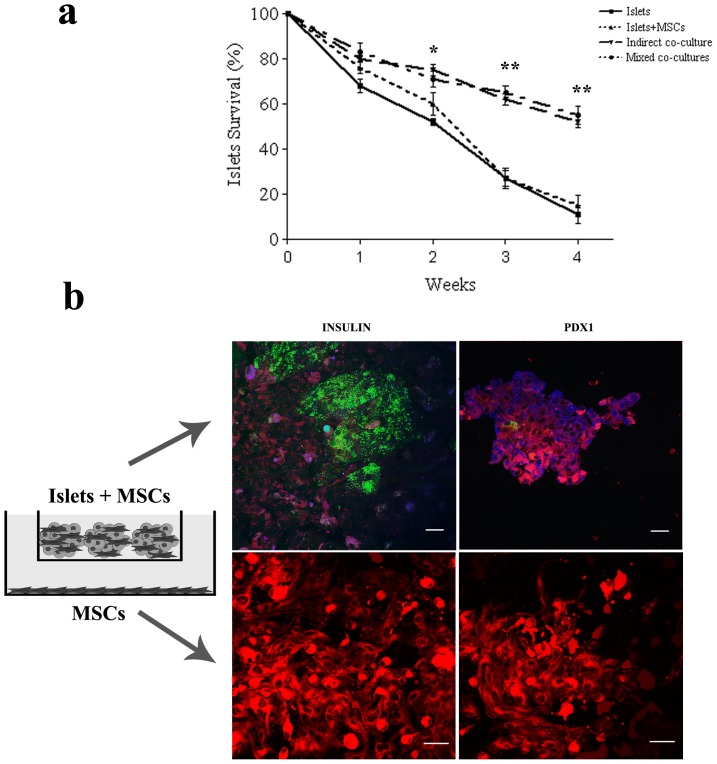
Mixed co-culture. Survival percentage evaluation of pancreatic islets indirectly co-cultured with MSCs. Calcein-positive cells were counted and the survival percentage was calculated up to 4 weeks of culture. Results are expressed as mean ± SD of three independent experiments. *P<0.05 ** Mixed co-cultures vs Islets, *P*<0.001 Mixed co-cultures vs Islets and Mixed co-cultures vs Islets+MSCs. Insulin and Pdx1 positive cells in mixed co-cultures made up of pancreatic islets coated with MSCs (within the Transwell, upper panel), and MSCs adhering to the cover glass (lower panel). In green, calcein positive pancreatic islets. In red, MSCs stained with DiI. In blue, insulin or Pdx1 positive cells. Bar 30 µm.

In order to verify if the two distinct positive effects exerted by direct and indirect co-cultures could co-exist, mixed co-cultures were set up in which MSCs were both in direct contact with pancreatic islets in a Transwell, and adherent to the bottom of the dish. In these kinds of cultures an increase in pancreatic viability was observed, similar to that reported for indirect co-cultures (Fig. 6a). Moreover, the immunofluorescence detection for insulin and Pdx1 expression evidenced the presence of these proteins in MSCs touching the islets, while these markers were absent in MSCs coating the dish (Fig. 6b), thus achieving the goal of uniting the distinct mechanisms of action in a single paradigm.

## Discussion

In this paper we demonstrated that MSCs, which have initially been used in combination with pancreatic islets for their immunosuppressive properties, are able to affect both pancreatic islets' survival and functionality by acting through different mechanisms, depending on the culture conditions. The direct contact between MSCs and pancreatic islets was able to trigger the differentiation of MSCs into insulin-releasing cells, rather than promoting islets' survival. On the contrary, in the indirect co-cultures the effect was an increase in islets' survival. This effect was probably mediated by the trophic factors, such as VEGF [15], Von Willebrand factor [14]–[17], and Il-6 [20], released by MSCs.

A “true” direct co-culture in which the MCSs were in contact with pancreatic islets was difficult to achieve. MSCs, simply added to the culture, can grow in adhesion to floating pancreatic islets, thus preserving both the tridimensional structure of pancreatic islets (and, therefore, their properties), and the correct culture conditions of MSCs, a basic requisite for maintaining their potential. It is important to maintain pancreatic islets in suspension since, when adherent to a substrate, they lose their particular phenotype and secretion properties [28]–[30]. The validity of this kind of culture has already been demonstrated by Duprez et al. [31], but only for a short period of culture. Our results, however, demonstrated that MSCs adhere to floating pancreatic islets for a period of at least 4 weeks.

The increased insulin level released into the culture medium of direct co-cultures has been already reported by several authors in vitro [27], [32]. *In vivo* studies have demonstrated that, when transplanted with MSCs, a lower number of pancreatic islets is necessary to achieve normoglycemia in diabetic rats [14]–[16]. The insulin increase is generally ascribed to an improved functionality of pancreatic islets mediated by MSCs. Our observations, however, demonstrated that MSCs differentiate into insulin-releasing cells, thereby increasing the amount of secreted insulin in the culture medium, thus offering an explanation of the results reported in *in vivo* studies. The differentiation of MSCs into insulin-releasing cells was exclusively obtained when MSCs and pancreatic islets were in contact. Among the genes activating the endocrine pathways, Pdx1, a pivotal factor for insulin production, has been shown to be involved. In the literature MSCs' differentiation into insulin-releasing cells [21]–[25], has been achieved by exposure to chemical factors [21], [22] or by genetic manipulations with the Pdx1 gene [23]–[25]. In our paradigm the simple contact of pancreatic islets with MSCs is sufficient to trigger the intracellular pathway which leads to insulin production, without the need for potentially dangerous procedures.

In turn, MSCs are able to affect pancreatic islets' behavior, but through some different mechanisms. The MSC coating improves the health condition of pancreatic islets, probably by preserving membrane integrity [27] and by releasing trophic factors [14]–[16], while indirect co-cultures are able to increase islets' survival, but not insulin release. These results are basically different from those obtained by Jung et al. [27] who observed increased islet survival only in direct co-cultures with MSCs. This difference may be ascribed to the different culture set up: in Jung and colleagues' paradigm the direct co-cultures are characterized by the presence of MSCs both on the islets' membrane and adhering to the bottom of the dish. However, this condition brings adherent MSCs to sequestrate pancreatic islets, thus hampering the floating state and giving a two dimensional islet culture, no longer in suspension but adhering to the dish. Moreover, this set up ensures that a great number of MSCs is present in the culture, both in direct contact with pancreatic islets and adhering to the dish being, therefore, more similar to our “mixed co-cultures”. In our “direct co-culture” model the MSCs present in the culture are fewer with respect to Jung's study, all of them being attached to pancreatic islets, probably insufficient to give a prolonged survival, but the close contact with pancreatic islets drives MSCs to enter into a differentiative pathway. In our indirect co-culture model, on the contrary, the MSCs are more numerous than in the direct one. MSCs reach confluence on the dish, and are unable to directly interact with pancreatic islets and vice versa, but are still able to release trophic factor allowing a prolonged islet survival.

Some trophic factors released by MSCs promoting islet survival have already been extensively described in several studies [14],[15],[19],[20],[27]. In particular, VEGF, Von Willebrandt and Il-6 are known to be released by MSCs also after co-culturing with pancreatic islets. Here, further investigation was carried out into the role of the poorly investigated CNTF, a trophic factor that is able to increase islet viability when exogenously administered [18], and also known to be expressed by MSC cultures [17]. In our study, we found that CNTF was expressed in pancreatic islets as well as in direct, indirect and mixed co-cultures, but its release was so low as to be undetectable in all the samples. For this reason, we hypothesize that CNTF is not involved in islet survival promotion by MSCs, but is most probably mediated by the trophic soluble factors already described in the literature [14], [15], [19], [20], [27].

It is indeed evident that, depending on the co-culture paradigms and through various mechanisms, MSCs are able to strengthen the therapeutic potential of pancreatic islet transplantation for the management of diabetes mellitus. MSCs can: i) directly contribute to insulin production through a differentiation process; ii) increase the survival of pancreatic islets. As we have demonstrated, in mixed co-cultures both the distinct mechanisms co-exist, thus representing the best model for increasing islet transplantation's therapeutic potential.

The co-transplantation of MSCs with pancreatic islets is a promising prospect for type 1 diabetes treatment, and more in-depth knowledge of the mechanisms involved in MSCs' activity may help to further improve the feasibility of this therapeutic option and its effectiveness.
